# Three-party authenticated key agreements for optimal communication

**DOI:** 10.1371/journal.pone.0174473

**Published:** 2017-03-29

**Authors:** Tian-Fu Lee, Tzonelih Hwang

**Affiliations:** 1 Department of Medical Informatics, Institute of Medical Sciences, Tzu Chi University, Hualien, Taiwan, ROC; 2 Department of Computer Science and Information Engineering, National Cheng Kung University, Tainan, Taiwan, ROC; King Saud University, SAUDI ARABIA

## Abstract

Authenticated key agreements enable users to determine session keys, and to securely communicate with others over an insecure channel via the session keys. This study investigates the lower bounds on communications for three-party authenticated key agreements and considers whether or not the sub-keys for generating a session key can be revealed in the channel. Since two clients do not share any common secret key, they require the help of the server to authenticate their identities and exchange confidential and authenticated information over insecure networks. However, if the session key security is based on asymmetric cryptosystems, then revealing the sub-keys cannot compromise the session key. The clients can directly exchange the sub-keys and reduce the transmissions. In addition, authenticated key agreements were developed by using the derived results of the lower bounds on communications. Compared with related approaches, the proposed protocols had fewer transmissions and realized the lower bounds on communications.

## Introduction

Authenticated key agreements (AKA) enable users to exchange confidential and authenticated information over an insecure network, and to establish a common key that can be employed to encrypt all communications over an insecure channel. In an AKA protocol, each communicating entity that wants to determine session keys is assured of the identity of each of the others to provide mutual authentication. In terms of realizing mutual authentication, AKA protocols can be divided into two types—implicit mutual authentication and explicit mutual authentication. An AKA protocol with implicit mutual authentication realizes mutual authentication in later communications. However, it is not possible to be certain how protocol participants will use the session key. In contrast, an AKA protocol with explicit mutual authentication (AKA-MA) realizes mutual authentication while executing the protocol [1].

The AKA protocols mainly focus on providing higher security and developing transmission efficiency. Numerous factors influence transmission efficiency. Aside from the computational complexity of an authentication protocol, message efficiency and round efficiency are two important evaluation criteria. Message efficiency considers the number of messages required to complete the protocol. A message is a data item sent from one party to a single destination at a particular time. Round efficiency considers the number of rounds required to complete the protocol. A round comprises all of the independent messages that can be sent and received in parallel [2,3].

Three-party authenticated key agreement (3AKA) protocol enables two users to agree a common session key for establishing a secure channel via the help of a trusted server. Recently, several approaches involving 3AKA-MA protocols have been presented. For instance, Gong et al. [2–4] provided lower bounds on communications for 3AKA-MA, which required five messages and four rounds. They also developed 3AKA-MA protocols to realize these lower bounds [2–4]. Kwon et al. [5–8] presented password-based 3AKA-MA protocols. In addition, some 3AKA-MA approaches have modified the structures of session keys to ensure perfect forward secrecy. For instance, the 3AKA-MA protocols in [3–13] based on the Diffie-Hellman problem [14] could provide perfect forward secrecy. Lee et al. [15] developed a 3AKA-MA based on chaotic maps without password table. Amin et al. [16] proposed anonymity preserving three-factor authenticated key exchange protocol for wireless sensor network. With reference to transmission, all of the 3AKA-MA protocols described above and other related secure approaches [14, 17–26] involve at least five messages or four rounds.

For 3AKA-MA protocols, few studies on the lower bounds on communication have been presented up to now, except for the investigation of Gong in [2,3]. However, Gong only considered this issue for conventional 3AKA-MA protocols, without ever completely discussing 3AKA-MA protocols. In 3AKA-MA protocols, two clients do not share any common secret key. Thus, they require the help of the server to authenticate the participants′ identities and exchange confidential and authenticated information over an insecure network. In conventional 3AKA-MA protocols, the sub-keys for generating a session key cannot be revealed in the channel. Clients must exchange their sub-keys with the help of the server to establish an authentication key (session key). Accordingly, a conventional 3AKA-MA protocol requires at least five messages and four rounds [2, 3]. However, if the session key is based on asymmetric cryptosystems, such as the Diffie-Hellman key exchange or the Elliptic Curve Diffie-Hellman key exchange, then revealing the sub-keys for generating the session key cannot compromise the session key. The clients can directly exchange the sub-keys without using the server, and thus the number of messages and rounds can be reduced.

This study investigated the rules according to the behavior patterns of AKA-MA protocols, and then derived the lower bounds of communications for 3AKA-MA protocols based on these rules. In addition, we used the derived results to develop communication-efficient 3AKA-MA protocols, including conventional 3AKA-MA protocols whose sub-keys cannot be revealed in the channel and 3AKA-MA protocols in which revealing the sub-keys cannot compromise the session key. The proposed conventional 3AKA-MA protocols require five messages and four rounds of communication and realize the lower bounds on the number of messages and rounds for conventional 3AKA-MA protocols. On the other hand, in the proposed 3AKA-MA protocols, the session key security is based on the Diffie-Hellman problem [14]. Revealing the information *g*^*x*^ mod *p* and *g*^*y*^ mod *p* for generating the session key (*g*^*xy*^ mod *p*) cannot compromise the session key itself because the session key cannot be determined without a knowledge of *x* or *y*, where *p* is a large prime. Therefore, the clients can publicly exchange the information *g*^*x*^ mod *p* and *g*^*y*^ mod *p* for generating the session key without the help of the server. Using this technique, the proposed protocol reduced the number of messages and rounds and required only four messages and three rounds of communications. Hence, the proposed 3AKA-MA protocol also realized the proposed lower bounds on the number of messages and rounds for 3AKA-MA protocols. Furthermore, the proposed 3AKA-MA protocols were proven secure [27–31] and have AKE security and MA security. Compared with related 3AKA-MA protocols, the proposed protocols were more efficient in communications, realized the lower bounds on the number of messages and rounds for 3AKA-MA protocols, and were suitable for practical environments.

This study is organized as follows. Section 2 describes the underlying primitives used in this investigation. Section 3 derives and proves the lower bounds on messages and rounds for 3AKA-MA protocols. Section 4 develops communication-efficient 3AKA-MA protocols based on the derived results from Section 3. All of the proposed protocols realize the lower bounds on the number of messages and rounds of communications. Section 5 provides security analyses and compares the performance of the proposed 3AKA-MA protocols with related protocols. Finally, Section 6 draws conclusions.

## Preliminaries

This section describes the underlying primitives used in this paper. The underlying primitives include session key security, mutual authentication security, the authenticator, the chosen ciphertext secure symmetric-key encryption, the Diffie-Hellman assumptions, and the cryptographic hash functions.

### AKE security (session key security)

In this security definition, the adversary is allowed to ask many ***Test*** queries as it wants. If a ***Test*** query is asked to a client instance that has not *accepted*, then return the invalid symbol ⊥. If a ***Test*** query is asked to an instance of an honest participant whose intended partner is dishonest or to an instance of a dishonest participant, then returns the real session key. Otherwise, the ***Test*** query decides to return either the real session key or a random string via an unbiased coin *c*. The adversary aims to correctly guess the value of the hidden bit *c* used by the Test oracle. Let *E* denote the event that the adversary wins this game. The *ake-advantage* of the event that an adversary violates the indistinguishability of the protocol **P** $Adv_{P}^{ake}(A)$. The protocol **P** is AKE-secure if $Adv_{P}^{ake}(A)$ is negligible. [27]

### Mutual Authentication (MA) security

In executing protocol **P**, the adversary A violates mutual authentication if A can fake the authenticator *μ*_*A*_ or *μ*_*B*_. The probability of this event is denoted by $Adv_{P}^{ma}(A)$. The protocol **P** is MA-secure if $Adv_{P}^{ma}(A)$ is negligible.

### Authenticator

Additional information appended to a message to enable the receiver to verify that the message should be accepted as authentic. For AKA-MA protocols, an authenticator is used for the receiver to assure that the sender has the common session key. [32]

### Chosen ciphertext secure symmetric-key encryption

For a symmetric-key encryption scheme, the CCA-advantage of the adversary A is the probability that A breaks the indistinguishability under Chosen Ciphertext Attacks, and denoted by $Adv_{sk}(A)$. The symmetric-key encryption scheme *SE* is Chosen Ciphertext Secure if $Adv_{sk}(A)$ is negligible [30].

### Decisional Diffie-Hellman (DDH) assumption

Let *G* = 〈*g〉* be a cyclic group of prime order *q* and *x*, *y*, *z* are randomly chosen in *Z*_*q*_. A *DDH* attacker A, a probabilistic Turing Machine, is defined as follows: Using the value of a random bit *c* decides the value of *Z*, which is *g*^*xy*^ mod *p* if *c* = 1 and *g*^*z*^ mod *p* if *c* = 0 where, *p* is a large prime. Given (*X*, *Y*, *Z*), A can correctly guess the bit *c* with probability $Adv_{G}^{ddh}(A)$ within polynomial time *t*. The Decisional Diffie-Hellman Assumption states that for every probabilistic polynomial time Turing Machine A, for large enough *k*, $Adv_{G}^{ddh}(A)\leq \varepsilon \left(k\right)$, where *ε*(*k*) is a negligible function.

## Lower bounds on number of messages and rounds for three-party AKA-MA protocols

This section first introduces the rules according to the behavior patterns of AKA-MA protocols, and then derives the lower bounds on the number of messages and rounds for three-party AKA-MA protocols based on these rules. The rules for AKA-MA protocols are as follows.

### The rules for AKA-MA protocols

**Rule 1**. In the AKA-MA protocol, the originator is the only one who initiates a message. The others can issue messages only at the moment they receive one. The protocol will proceed in sequential order.
**Rule 2**. In the AKA-MA protocol, each participant has to send out a message.
**Rule 3**. In the AKA-MA protocol, to derive a session key a client has to receive messages from all other clients.
**Rule 4**. In the AKA-MA protocol, a client cannot issue an authenticator before it derives the session key.
**Rule 5**. In the AKA-MA protocol, each client must send out an authenticator, and receive and verify authenticators from all other clients.
In the 3AKA-MA protocol, the clients do not share any secret key and thus authenticate each other via the help of the trusted server.
**Rule 6**. In the 3AKA-MA protocol, client A can only authenticate B via S, and vice versa.
**Rule 7**. In the 3AKA-MA protocol, if a sub-key cannot be revealed in the channel, then client A can only obtain the sub-key from B via S, and vice versa.

### Lower bounds on number of messages and rounds for three-party AKA-MA protocols

This subsection provides the lower bounds on the number of messages and rounds for the 3AKA-MA protocols, based on the rules described in Section 3.1.

**Theorem 1**. *Every 3AKA-MA protocol is implemented in at least five messages if the sub-keys cannot be revealed in the channel*.

**Fig 1:**
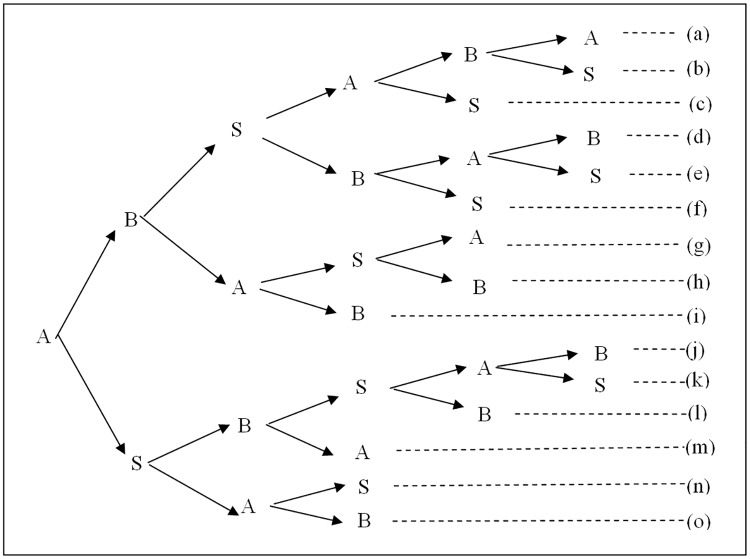
The branches for 3AKA-MA protocols if the sub-keys cannot be revealed in the channel by Rules 1 and 2.

**Proof:** In a 3AKA-MA protocol, by Rule 1, the protocol originator A initiates a message, the other participants B and S issue messages at the moment of receiving one, and the protocol proceeds in sequential order. If the sub-keys cannot be revealed in the channel, then we have many variable protocols, as shown in Fig 1.

For protocol (a), after the third message, A receives a message sent from B and transmitted by S. A can authenticate B via S by Rule 6, receive a sub-key *K*_2_ from B, and derive the session key SK by Rules 3 and 7. Then, by Rule 4, A can construct and issue an authenticator *auth*_*A*_ in the fourth message. On the other hand, after the fourth message, B receives a message sent from B and transmitted by S. Similarly, B can authenticate A via S, receive a sub-key *K*_1_ from A, and derive the session key SK. Then, by Rule 4, B can verify *auth*_*A*_ from A, and construct and issue an authenticator *auth*_*B*_ in the fifth message. Finally, A can verify *auth*_*B*_ from B. Hence, for A and B, five messages are required. In addition, for S, three messages are required by Rules 1 and 2. Therefore, the 3AKA-MA protocol can be implemented in five messages in (a).

For protocol (b), after the fourth message, B can receive a sub-key *K*_1_ sent from A and transmitted by S. Then B can derive the session key SK and issue an authenticator *auth*_*B*_ in the fifth message by Rules 3, 4, and 7. However, by Rule 5, A must receive and verify *auth*_*B*_ from B. For A, at least one extra message is required. Thus protocol (b) cannot be implemented in five messages.

For protocol (c), by Rules 3 and 7, B cannot derive the session key SK until it receives a sub-key *K*_1_ sent from A and transmitted by S. Thus, for B, an extra message is required to receive the sub-key *K*_1_ from A. In addition, another extra one is required to issue an authenticator *auth*_*B*_ by Rules 4 and 5. Hence, at least two extra messages are required. Thus protocol (c) cannot be implemented in five messages.

For protocol (d), after the third message, B can authenticate A via S by Rule 6, receive a sub-key *K*_1_ sent from A and transmitted by S, and derive the session key SK by Rules 3 and 7. Then, by Rule 4, B can compute and issue an authenticator *auth*_*B*_ in the fourth message. Similarly, after the fourth message, A can authenticate B via S by Rule 6, receive a sub-key *K*_2_ sent from B and transmitted by S, and derive the session key SK by Rules 3 and 7. Then, by Rule 4, A can verify *auth*_*B*_ from B, and compute and issue an authenticator *auth*_*A*_ in the fifth message. Finally, B can verify *auth*_*A*_ from A. Hence, for A and B, five messages are required. In addition, for S, three messages are required by Rules 1 and 2. Therefore, the 3AKA-MA protocol can be implemented in five messages in (d).

For protocol (e), after the fourth message, A receives a message sent from B and transmitted by S. A can receive a sub-key *K*_2_ from B and derive the session key SK by Rules 3 and 7. By Rule 4, A can issue an authenticator *auth*_*A*_ in the fifth message. However, B must receive and verify *auth*_*A*_ from A by Rule 5. For B, at least one extra message is required. Thus, protocol (e) cannot be implemented in five messages.

For protocol (f), by Rules 3 and 7, A must receive a message that includes a sub-key *K*_2_ sent from B and transmitted by S. Then A can derive the session key SK. Thus, for A, an extra message is required to receive the sub-key *K*_2_ from B and another extra message is required to issue an authenticator *auth*_*A*_ by Rules 4 and 5. Hence, at least two extra messages are required. Thus the protocol (f) cannot be implemented in five messages.

For protocol (g), from arguments similar to protocol (b), at least two extra messages are required. Thus, protocol (g) cannot be implemented in five messages.

For protocol (h), from arguments similar to protocol (f), at least two extra messages are required. Thus, protocol (h) cannot be implemented in five messages.

For protocol (i), by Rules 3 and 7, A cannot derive the session key SK until it receives a message that includes a sub-key *K*_2_ sent from B and transmitted by S. Thus, for A, two extra messages are required to receive sub-key *K*_2_ from B and another extra one is required to issue an authenticator *auth*_*A*_ by Rules 4 and 5. Hence, at least three extra messages are required. Thus, protocol (i) cannot be implemented in five messages.

For protocol (j), after the second message, B can authenticate A via S by Rule 6, receive a sub-key *K*_1_ sent from A and transmitted by S, and derive the session key SK by Rules 3 and 7. Then, from arguments similar to protocol (d), five messages are required for A and B and four messages are required for S. Therefore, the 3AKA-MA protocol can be implemented in five messages in (j).

For protocol (k), from arguments similar to protocol (e), at least one extra message is required. Thus, protocol (k) cannot be implemented in five messages.

For protocol (l), from arguments similar to protocol (f), at least two extra messages are required. Thus, protocol (l) cannot be implemented in five messages.

For protocols (m) and (n), from arguments similar to protocol (i), at least three extra messages are required. Thus, protocols (m) and (n) cannot be implemented in five messages.

For protocol (o), by Rules 1 and 2, B can send out a message that includes sub-key *K*_2_ only after receiving one, and must send out a message. Therefore, at least two extra messages are required. In addition, A cannot derive the session key SK until it receives sub-key *K*_2_ sent from B and transmitted by S. Thus, for A, an extra message is required to receive the sub-key *K*_2_ transmitted by S. In addition, another extra one is required to issue an authenticator *auth*_*A*_ by Rules 4 and 5. Hence, at least three extra messages are required. Thus, protocol (o) cannot be implemented in five messages.

Table 1 summarizes the analyses of protocols (a), (b),…, (n) and (o). From these analyses, we can conclude that, with the exceptions of protocols (a), (d), and (j), these 3AKA-MA protocols cannot be implemented in five messages. Therefore, every 3AKA-MA protocol requires at least five messages for implementation if the sub-keys cannot be revealed in the channel.

**Table 1:** The messages of 3AKA-MA protocols are required in communications if the sub-keys cannot be revealed in the channel.

Protocols	(a)	(b)	(c)	(d)	(e)	(f)	(g)	(h)
Required messages	5	≥ 6	≥ 6	5	≥ 6	≥ 6	≥ 6	≥ 6
Protocols	(i)	(j)	(k)	(l)	(m)	(n)	(o)	
Required messages	≥ 6	5	≥ 6	≥ 6	≥ 6	≥ 6	≥ 6	

**Theorem 2**. *Every 3AKA-MA protocol is implemented in at least four rounds if the sub-keys cannot be revealed in the channel*.

**Proof:** In a 3AKA-MA protocol, by Rule 1, the protocol originator A initiates a message including a sub-key *K*_1_ for generating a session key to the trusted server S in the first round. Then, in the second round, S forwards the message including *K*_1_ to B. At the same time, B sends a message including a sub-key *K*_2_ to S. After receiving the message including *K*_1_ from S, B can authenticate A by Rule 6 and derive the session key *SK* by Rules 3 and 7. In the third round, S forwards the message including a sub-key *K*_2_ to A, and B can construct and issue an authenticator *auth*_*B*_ to A by Rule 4. After receiving the messages including *K*_2_ transmitted by S and *auth*_*B*_ from B, A can authenticate B via S by Rule 6, derives a session key *SK* by Rules 3 and 7, and verifies *auth*_*B*_ from B. Then, in the fourth round, A can compute and issue an authenticator *auth*_*A*_ by Rule 4. Finally, B verifies *auth*_*A*_ from A. Hence, by Rule 5, every 3AKA-MA protocol is implemented in at least four rounds if the sub-keys cannot be revealed in the channel.

**Theorem 3**. *Every 3AKA-MA protocol is implemented in at least four messages*.

**Fig 2:**
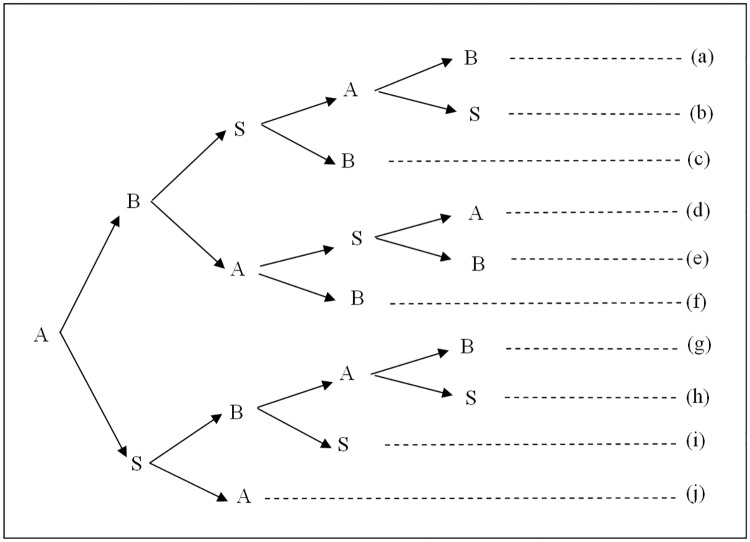
The branches for 3AKA-MA protocols by Rules 1 and 2.

By using similar arguments in Theorem 1, we have many variable protocols, as shown in Fig 2, and have Table 2, which summarizes the analyses of protocols (a),(b),…, (i) and (j). We also can conclude that with the exceptions of protocol (a), these 3AKA-MA protocols cannot be implemented in four messages. Therefore, every 3AKA-MA protocol requires at least four messages for implementation.

**Table 2:** The number of messages for 3AKA-MA protocols are required in communications.

Protocol	(a)	(b)	(c)	(d)	(e)
Required messages at least	4	≥ 5	≥ 5	≥ 5	≥ 5
Protocol	(f)	(g)	(h)	(i)	(j)
Required messages at least	≥ 5	≥ 5	≥ 6	≥ 5	≥ 5

**Theorem 4**. *Every 3AKA-MA protocol is implemented in at least three rounds*.

**Proof:** In a 3AKA-MA protocol, by Rule 1, the protocol originator A initiates messages for authentication and including a sub-key *K*_1_ in the first round. After receiving the message including *K*_1_, B can randomly select a sub-key *K*_2_ and compute the session key *SK* by Rule 3 and an authenticator *auth*_*B*_ by Rules 3 and 4. In the second round, S sends the message to B for authenticating A, so that B can authenticate A via S by Rule 6. In addition, B also sends out messages for authentication and including *K*_2_ and *auth*_*B*_. After receiving *K*_2_ and *auth*_*B*_ from B, A can derive a session key *SK* by Rules 3. Then A can verify *auth*_*B*_, compute an authenticator *auth*_*A*_ by Rule 4, and send a message including *auth*_*A*_ to B in the third round. Simultaneously, S sends out messages so that A can authenticate B via S by Rule 6 and B can verify *auth*_*A*_. Hence, by Rule 5, every 3AKA-MA protocol is implemented in at least four rounds.

This section has provided the lower bounds on the number of messages and rounds for the 3AKA and 3AKA-MA protocols based on the rules described in Section 3.1. In the next section, we will present 3AKA-MA protocols that realize the lower bounds on the number of messages and rounds for 3AKA-MA protocols based on the results of above theorems.

## The communication-efficient 3AKA-MA protocols

This section will use the derived communication results of Section III to develop secure and communication-efficient 3AKA-MA protocols. First, we present 3AKA-MA protocols whose sub-keys cannot be revealed in the channel, and then propose 3AKA-MA protocols in which revealing the sub-keys cannot compromise the session key.

Assume that *A* and *B* are two communicating parties and that *S* is a trusted server. Clients *A* and *B* share long-lived keys *K*_*AS*_ and *K*_*BS*_ respectively, with server *S*. The notation used throughout this section is as follows:

Notation

*p*, *g*: A large prime *p* and a generator *g* in group $Z_{p}^{*}$, a group in which the Diffie-Hellman problem is considered hard.
*x*, *y*: Random exponents chosen by *A* and *B*.
{*M*}_*K*_: Encryption of *M* using a symmetric encryption scheme with a cryptographically strong shared key *K*.
*H*(*M*): A one-way hash function *H* applied to *M* [32].
*M*_1_, *M*_2_: *M*_1_ is concatenated with *M*_2_.
*A* → *B*: *M*: *A* sends message *M* to *B*.

### The communication-efficient nonce-based 3AKA-MA protocols for cases where the sub-keys cannot be revealed in the channel

In the proposed 3AKA-MA protocols, *A* and *B* randomly select sub-keys *K*_1_ and *K*_2_, respectively. Since the sub-keys cannot be revealed in the channel, *A* obtains the sub-key *K*_2_ from B via S, and vice versa. Then, they can derive a common session key *SK* ≡ *f*(*K*_1_, *K*_2_). Finally, they compute and send out their authenticators *μ*_*A*_ and *μ*_*B*_. All of the proposed 3AKA-MA protocols were developed based on Theorem 1 and Theorem 2 in Section 3 and executed using five messages and four rounds.

#### Proposed message-efficient nonce-based 3AKA1-MA protocol

**Fig 3:**
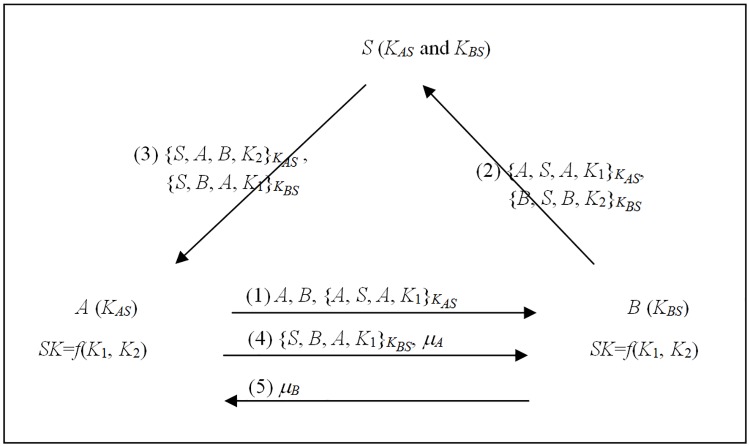
The message-efficient 3AKA1-MA protocol.

The proposed message-efficient nonce-based 3AKA1-MA protocol was developed based on protocol (a) in Theorem 1 for a case where the sub-keys cannot be revealed in the channel. Fig 3 depicts the proposed 3AKA1-MA protocol, which will now be described in detail.

1. A→B: $A,B,{\left\{A,S,A,K_{1}\right\}}_{K_{AS}}$
  *A* selects a random number *K*_1_ as the sub-key, encrypts (*A*, *S*, *A*, *K*_1_) using *A*'s secret key *K*_*AS*_, and sends $A,B,{\left\{A,S,A,g^{N_{A}}\right\}}_{K_{AS}}$ to *B*.
2. B→S:$A,B,{\left\{A,S,A,K_{1}\right\}}_{K_{AS}}$, ${\left\{B,S,B,K_{2}\right\}}_{K_{BS}}$
  Similarly, *B* selects a random number *K*_2_ as the sub-key, encrypts (*B*, *S*, *B*, *K*_2_) using *B*'s secret key *K*_*BS*_. Then it sends ${\left\{B,S,B,K_{2}\right\}}_{K_{BS}}$ and forwards $A,B,{\left\{A,S,A,g^{N_{A}}\right\}}_{K_{AS}}$ to *S*.
3. S→A: ${\left\{S,A,B,K_{2}\right\}}_{K_{AS}}$, ${\left\{S,B,A,K_{1}\right\}}_{K_{BS}}$
  *S* decrypts ${\left\{A,S,A,K_{1}\right\}}_{K_{AS}}$ and ${\left\{B,S,B,K_{2}\right\}}_{K_{BS}}$ with secret keys *K*_*AS*_ and *K*_*BS*_, and authenticates *A* and *B* by checking *A*′s ID and *B*′s ID, respectively. If it is successful, *S* then sends ${\left\{S,A,B,K_{2}\right\}}_{K_{AS}}$ and ${\left\{S,B,A,K_{1}\right\}}_{K_{BS}}$ to *A*.
4. A→B: ${\left\{S,B,A,K_{1}\right\}}_{K_{BS}}$, *μ*_*A*_
  *A* obtains *K*_2_ by decrypting ${\left\{S,A,B,K_{2}\right\}}_{K_{AS}}$ with *K*_*AS*_ and derives the session key *SK* ≡ *f*(*K*_1_, *K*_2_). Then it computes an authenticator *μ*_*A*_ = *H*(*A*, *B*, *SK*), and sends ${\left\{S,B,A,K_{1}\right\}}_{K_{BS}}$ and *μ*_*A*_ to *B*.
5. B→A: *μ*_*B*_
  Similarly, *B* obtains *K*_1_ by decrypting ${\left\{S,B,A,K_{1}\right\}}_{K_{BS}}$ with *K*_*BS*_ and derives the session key *SK* ≡ *f*(*K*_1_, *K*_2_). If *B* successfully verifies *μ*_*A*_ from *A*, then it computes an authenticator *μ*_*B*_ = *H*(*B*, *A*, *SK*) and sends *μ*_*B*_ to *A*. Finally, *A* can verifies *μ*_*B*_ from *B*. Accordingly, *A* and *B* have the common session key *SK* ≡ *f*(*K*_1_, *K*_2_).

#### Proposed message-efficient nonce-based 3AKA2-MA protocol

**Fig 4:**
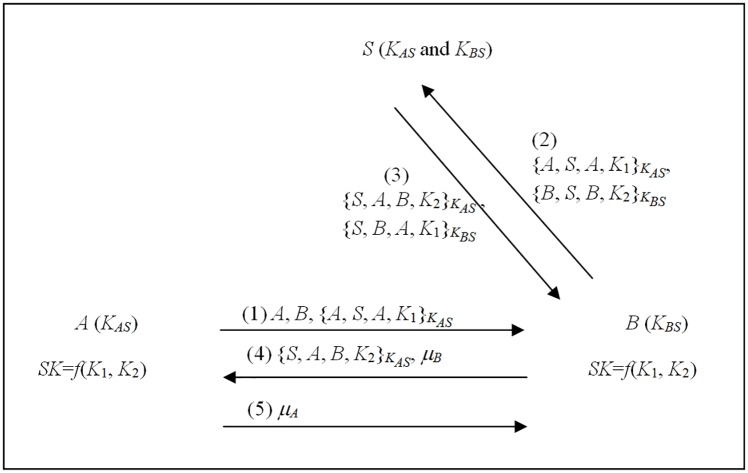
The message-efficient 3AKA2-MA protocol.

The proposed message-efficient nonce-based 3AKA2-MA protocol was developed based on protocol (d) in Theorem 1 for a case where the sub-keys cannot be revealed in the channel. Fig 4 depicts the proposed 3AKA2-MA protocol, which is described as follows.

1. A→B: $A,B,{\left\{A,S,A,K_{1}\right\}}_{K_{AS}}$
2. B→S: $A,B,{\left\{A,S,A,K_{1}\right\}}_{K_{AS}}$, ${\left\{B,S,B,K_{2}\right\}}_{K_{BS}}$
3. S→B: ${\left\{S,A,B,K_{2}\right\}}_{K_{AS}}$, ${\left\{S,B,A,K_{1}\right\}}_{K_{BS}}$
4. B→A: ${\left\{S,A,B,K_{2}\right\}}_{K_{AS}}$, *μ*_*B*_
5. A→B: *μ*_*A*_

#### Proposed message-efficient nonce-based 3AKA3-MA protocol

**Fig 5:**
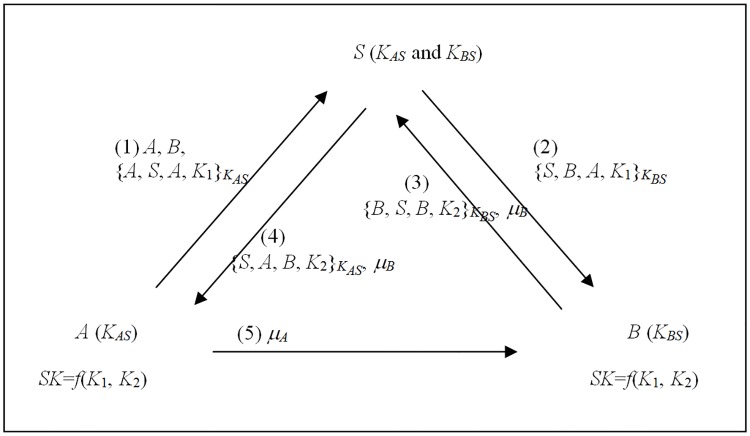
The message-efficient 3AKA3-MA protocol.

The message-efficient nonce-based 3AKA3-MA protocol was developed based on protocol (j) in Theorem 1 for a case where the sub-keys cannot be revealed in the channel. In fact, this 3AKA3-MA-3 protocol is the same as Gong′s nonce-based 3AKA-MA protocol in [2,3]. Fig 5 depicts the proposed 3AKA3-MA protocol, which is described as follows.

1. A→S: $A,B,{\left\{A,S,A,K_{1}\right\}}_{K_{AS}}$
2. S→B: ${\left\{S,A,B,K_{1}\right\}}_{K_{AS}}$
3. B→S: ${\left\{B,S,B,K_{2}\right\}}_{K_{BS}}$, *μ*_*B*_
4. S→A: ${\left\{S,A,B,K_{2}\right\}}_{K_{AS}}$, *μ*_*B*_
5. A→B: *μ*_*A*_

#### Proposed round-efficient nonce-based 3AKA-MA protocol

The proposed round-efficient nonce-based R3AKA-MA protocol was developed based on Theorem 2 and can be executed in four rounds. The proposed protocol is described as follows.

1. A→S: $A,B,{\left\{A,S,A,K_{1}\right\}}_{K_{AS}}$
  A→B: *A*, *B*
2. B→S: $A,B,{\left\{B,S,B,K_{2}\right\}}_{K_{BS}}$
3. S→A: ${\left\{S,A,B,K_{2}\right\}}_{K_{AS}}$
  S→B: ${\left\{S,B,A,K_{1}\right\}}_{K_{BS}}$
4. A→B: *μ*_*A*_
  B→A: *μ*_*B*_

### Proposed communication-efficient 3AKA-MA protocols for a case where revealing the sub-keys cannot compromise the session key

In an authenticated key agreement protocol, if the session key is based on asymmetric cryptosystems, such as the Diffie-Hellman key exchange or the Elliptic Curve (ECC) Diffie-Hellman key exchange [1, 33, 34], Chebyshev chaotic map-based Diffie-Hellman key exchange [15, 35, 36, 37] then revealing the sub-keys used to generate the session key cannot compromise the session key. This subsection will use the Diffie-Hellman key exchange to propose communication-efficient 3AKA-MA protocols in which revealing the sub-keys cannot compromise the session key.

In a Diffie-Hellman-based authentication protocol, revealing the sub-keys, *K*_1_ = *g*^*x*^ (mod *p*) and *K*_2_ = *g*^*y*^ (mod *p*), used to generate the session key (*SK* = *H(A*, *B*, *K*_1_, *K*_2_, *K*) cannot compromise the session key itself because the session key cannot be determined without a knowledge of *x* or *y*, where *K* ≡ *g*^*xy*^ (mod *p*). Therefore, clients *A* and *B* can publicly exchange the sub-keys *K*_1_ and *K*_2_ for generating the session key without the help of the server. Upon receiving *K*_1_ (or *K*_2_), the client can compute the session key and send out its authenticator *μ*_*B*_ = *H*(*B*, *A*, *K*_1_, *SK*) (or *μ*_*A*_ = *H*(*A*, *B*, *K*_2_, *SK*)). Finally, *A* and *B* can authenticate each other via *S*, have a common session key *SK*, and verify the authenticators *μ*_*B*_ and *μ*_*A*_.

#### Proposed message-efficient nonce-based DH-3AKA-MA protocol

**Fig 6:**
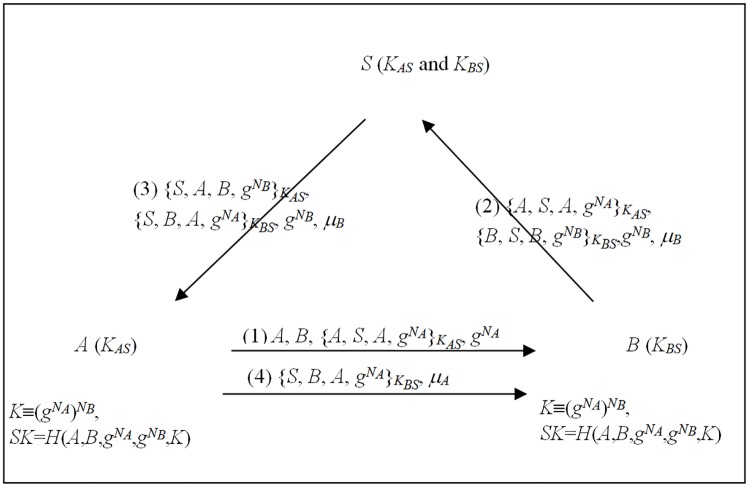
The message-efficient DH-3AKA-MA protocol.

The proposed message-efficient nonce-based DH-3AKA-MA protocol is developed according to the protocol (a) in Theorem 3. Fig 6 depicts the proposed DH-3AKA-MA protocol which will now be described in detail.

1. A→B: $A,B,{\left\{A,S,A,K_{1}\right\}}_{K_{AS}}$, *K*_1_
  *A* selects a random number *x*; computes the sub-key *K*_1_ = *g*^*x*^ (mod *p*) and encrypts (*A*, *S*, *A*, *K*_1_) using *A*'s secret key *K*_*AS*_. Then *A* sends $A,B,{\left\{A,S,A,K_{1}\right\}}_{K_{AS}}$, *K*_1_ to *B*.
2. B→S: $A,B,{\left\{A,S,A,K_{1}\right\}}_{K_{AS}}$, ${\left\{B,S,B,K_{2}\right\}}_{K_{BS}}$, *K*_2_, *μ*_*B*_
  Similarly, *B* selects random numbers *y*; computes the sub-key *K*_2_ = *g*^*y*^ (mod *p*) and encrypts (*B*, *S*, *B*, *K*_2_) using *B*'s secret key *K*_*BS*_. Simultaneously, *B* computes *K* ≡ (*K*_1_)^*y*^ (mod *p*), *SK* = *H*(*A*, *B*, *K*_1_, *K*_2_, *K*) as the session key shared with *A*, and an authenticator *μ*_*B*_ = *H*(*B*, *A*, *K*_1_, *SK*). Then *B* sends ${\left\{B,S,B,K_{2}\right\}}_{K_{BS}}$, *K*_2_, *μ*_*B*_ and forwards $A,B,{\left\{A,S,A,K_{1}\right\}}_{K_{AS}}$ to *S*.
3. S→A: ${\left\{S,A,B,K_{2}\right\}}_{K_{AS}}$, ${\left\{S,B,A,K_{1}\right\}}_{K_{BS}}$, *K*_2_, *μ*_*B*_
  *S* decrypts ${\left\{A,S,A,K_{1}\right\}}_{K_{AS}}$ and ${\left\{B,S,B,K_{2}\right\}}_{K_{BS}}$ with secret keys *K*_*AS*_ and *K*_*BS*_, and authenticates *A* and *B* by checking *A*′s ID and *B*′s ID, respectively. If it is successful, *S* then sends ${\left\{S,A,B,K_{2}\right\}}_{K_{AS}}$ and ${\left\{S,B,A,K_{1}\right\}}_{K_{BS}}$ as one-time certificates for *A* and *B*, respectively, and forwards *K*_2_, *μ*_*B*_ to *A*.
4. A→B: ${\left\{S,B,A,K_{1}\right\}}_{K_{BS}}$, *μ*_*A*_
  If *A* successfully validates *K*_2_ by decrypting ${\left\{S,A,B,K_{2}\right\}}_{K_{AS}}$ with *K*_*AS*_, then computes *K* ≡ (*K*_2_)^*x*^ (mod *p*), the session key *SK* = *H*(*A*, *B*, *K*_1_, *K*_2_, *K*), and an authenticator *μ*_*A*_ = *H*(*A*, *B*, *K*_2_, *SK*), and sends ${\left\{S,B,A,K_{1}\right\}}_{K_{BS}}$, *μ*_*A*_ to *B*. Finally, *B* authenticates *A* and *S* by decrypting ${\left\{S,B,A,K_{1}\right\}}_{K_{BS}}$ with *K*_*BS*_ and checking *K*_1_ and verifies the authenticator *μ*_*A*_ from *A*. Accordingly, *A* and *B* have a common session key *SK* = *H*(*A*, *B*, *K*_1_, *K*_2_, *K*).

#### Proposed round-efficient DH-R3AKA-MA protocol

The proposed round-efficient nonce-based DH-3AKA-MA (DH-R3AKA-MA) protocol is developed according to Theorem 4 and can be executed in three rounds. The proposed DH-3AKA-MA protocol is described as follows.

1. A→S: $A,B,{\left\{A,S,A,K_{1}\right\}}_{K_{AS}}$
  A→B: *A*, *B*, *K*_1_
2. B→S: $A,B,{\left\{B,S,B,K_{2}\right\}}_{K_{BS}}$
  B→A: *K*_2_, *μ*_*B*_
3. S→A: ${\left\{S,A,B,K_{2}\right\}}_{K_{AS}}$
  S→B: ${\left\{S,B,A,K_{1}\right\}}_{K_{BS}}$
  A→B: *μ*_*A*_

This section has presented four 3AKA-MA protocols whose sub-keys cannot be revealed in the channel, and two DH-3AKA-MA protocols in which revealing the sub-keys cannot compromise the session key. The proposed 3AKA1-MA, 3AKA2-MA, and 3AKA3-MA protocols require only five messages; the proposed R3AKA-MA protocol requires only four rounds; the proposed DH-3AKA-MA protocol requires only four messages; and the proposed DH-R3AKA-MA protocol requires only three rounds of communication. Thus all of the proposed 3AKA-MA protocols realize the lower bounds on the number of messages and rounds for 3AKA-MA protocols. In addition, we can construct synchronized clocks in a network environment and transform each of the proposed nonce-based 3AKA-MA protocols into a clock-based 3AKA-MA protocol by adding timestamps in ciphertexts. These obtained clock-based 3AKA-MA protocols do not require any extra message or round and thus also realize the lower bounds on the number of messages and rounds for 3AKA-MA protocols.

## Security and performance analyses

The proposed 3AKA-MA protocols and compares their performance with that of other related authentication protocols.

### Security proofs of proposed 3AKA-MA protocols

#### Communication model

**Protocol participants:** Two protocol participants *A* and *B* try to authenticate each other and establish an authentication key *SK* via the help of a trusted third party *S* in protocol **P**. A participant may be involved in numerous instances, called oracles, of distinct concurrent executions of **P**. The instance *i* of participant *U* is expressed as $\Pi_{U}^{i}$. [35]

**Long-lived keys:** The long-term secret key *K*_*AS*_ is shared between *A* and *S*, and the long-term secret key *K*_*BS*_ is shared between *B* and *S*. The long-lived keys *K*_*AS*_ and *K*_*BS*_ are defined as the symmetric keys of *A* and *B*, respectively.

**Oracle queries:** The following descriptions define oracle queries which model the capabilities of the adversary A.

- ***Send***$\left(\Pi_{U}^{i},M\right)$: In this query, the adversary A can control all communications in protocol **P**. When A sends oracle $\Pi_{U}^{i}$ a message *M*, $\Pi_{U}^{i}$ sends back the response message that is computed by executing **P**. A can send a user oracle $\Pi_{U}^{i}$ a query $\left(\Pi_{U}^{i},"start"\right)$ as initialization of executing **P** [35].
- ***Corrupt***(*U*): In this query, the adversary A who has compromised long-lived keys cannot compromise previous session keys. A sends a participant *U* this query, and returns *U*'s long-lived key.
- ***SymEnc ({E,D},k,{M,C})***: This query allows that adversary A accesses to encryption oracle SymEnc defined in previous section. When A sends SymEnc an encryption query ***SymEnc (E,k,M)***, SymEnc searches the **Γ***-table*. If a record (*k*,*M*,*C*) has been queried and recorded in the **Γ***-table*, SymEnc sends back the correspondent ciphertext *C*; otherwise SymEnc returns a random *C*, and appends (*k*,*M*,*C*) to the **Γ***-table*. Similarly, on receiving a decryption query ***SymEnc (D,k,C)***, if a record (*k*,*M*,*C*) has been queried and recorded in the **Γ***-table*, SymEnc sends backs the correspondent plaintext *M*; otherwise SymEnc returns a random *M*, and appends (*k*,*M*,*C*) to the **Γ**-*table*.
- ***Hash***(*M*): In this query, the adversary A receives hash results by sending a random oracle Ω queries. On receiving this query, if a record (*M*, *r*) has been queried and recorded in the **H***-table*, Ω send back *r*; otherwise Ω send back a random *r'*, and appends (*M*, *r'*) to the **H**-*table* [35].
- ***Reveal $\left(\Pi_{U}^{i}\right)$***: This query models known key attacks. The adversary A who has compromised one authentication key cannot reveal other authentication keys. The ***Reveal*** query is only available to adversary A when oracle $\Pi_{U}^{i}$ has accepted [35].
- ***Test***($\Pi_{U}^{i}$): This query measures the semantic security of the session key *SK*, which specifies the indistinguishability of the real session key from a random string. During the executing protocol **P**, adversary A can ask a single ***Test*** query at sometime. Upon receiving this query, $\Pi_{U}^{i}$ returns A the real session key *SK* or a random string by flipping an unbiased coin *c*. This query is available only when $\Pi_{U}^{i}$ is Fresh [35].

#### Security definitions

**Partnering:** Two user oracles $\Pi_{A}^{i}$ and $\Pi_{B}^{j}$ are partnered if

- oracles $\Pi_{A}^{i}$ and $\Pi_{B}^{j}$ directly exchange messages and
- only oracles $\Pi_{A}^{i}$ and $\Pi_{B}^{j}$ obtain the common session key *SK*.

**Freshness:** An oracle $\Pi_{U}^{i}$ is **Fresh** in **P** if.

- $\Pi_{U}^{i}$ has accepted *SK*, and
- $\Pi_{U}^{i}$ and its partner have not been sent a **Reveal** query.

#### Security proofs

The Difference Lemma [30] is used for our sequence of games and is described as follows:

**Lemma 5 (Difference Lemma)**. *Let A*, *B and F be events defined in some probability distribution*, *and suppose that A*∧¬*F* ⇔ *B*∧¬*F*. *Then*

|Pr[A]−Pr[B]|≤Pr[F].

The following theorem shows that the proposed 3AKA1-MA protocol has AKE security and provides mutual authentication by using the logical tool which was defined and presented by Burrows et al. [38] in 1990 and Buttyan et al. [39] in 1998.

**Theorem 6**. *The proposed 3AKA1-MA protocol has AKE security and provides mutual authentication*.

The proposed 3AKA1-MA, 3AKA2-MA, 3AKA3-MA, 3AKA4-MA protocols are similar with the proposed 3AKA1-MA protocol. These protocols reveal the same information in the channel. Their security proofs are almost the same, can be obtained by using similar arguments, and thus are not presented here.

In the following, we first prove AKE security of the DH-3PAKA protocol, which is transformed from the DH-3PAKA-MA protocol by removing the authenticators *μ*_*A*_ and *μ*_*B*_. Then, we use AKE security of the DH-3AKA protocol to prove AKE and MA securities of the DH-3PAKA-MA protocol. The DH-3AKA protocol is described as follow.

1. A→B: $A,B,{\left\{A,S,A,K_{1}\right\}}_{K_{AS}}$, *K*_1_
2. B→S: $A,B,{\left\{A,S,A,K_{1}\right\}}_{K_{AS}}$, ${\left\{B,S,B,K_{2}\right\}}_{K_{BS}}$, *K*_2_
3. S→A: ${\left\{S,A,B,K_{2}\right\}}_{K_{AS}}$, ${\left\{S,B,A,K_{1}\right\}}_{K_{BS}}$, *K*_2_
4. A→B: ${\left\{S,B,A,K_{1}\right\}}_{K_{BS}}$

The following theorem shows that the proposed DH-3AKA protocol has AKE security if the used long-term secret keys are secure and the Decisional Diffie-Hellman assumptions holds in G.

**Theorem 7**. *Let Adv*_*sk*_ *denote the advantage that an adversary breaks the long-term secret key within time t*_1_. *Let $Adv_{G}^{ddh}$ be the advantage that a DDH attacker solves the DDH problem within time t*_3_. *Then*, *the probability that an adversary breaks the AKE security of the DH-3AKA protocol*:

$$\begin{array}{l} Adv_{dh-3aka}^{ake}(t^{\prime },q_{\text{0}},q_{1},q_{2})\leq \frac{q_{1}{}^{2}+q_{2}{}^{2}}{2^{l-1}}+4\cdot Adv_{sk}(t_{\text{1}},q_{\text{0}},q_{1},q_{2})\\ \;+\text{2}\cdot Adv_{G}^{ddh}(t_{3},q_{\text{0}},q_{1},q_{2}), \end{array}$$

*where t*′ ≤ *t*_1_ *+ (q*_1_ +*q*_2_)⋅*τ*_1_ + 4⋅*τ*_3_;*q*_0_ *denotes the numbers of the Send queries; q*_1_ *and q*_2_ *denote the numbers of the SymEnc queries involving A and S*, *and involving B and S*, *respectively; l is a security parameter* [40]; *t*′ = *t*_1_ *+ (q*_1_ +*q*_2_)*τ*_1_; *and τ*_1_ *is the time to compute a symmetric en/decryption; and τ*_3_ *is the time to perform an exponential computation*.

The following theorem shows that the proposed DH-3AKA-MA protocol has AKE security if the used hash function is secure and the DH-3AKA protocol has AKE security.

**Theorem 8**. *Let $Adv_{dh-3aka}^{ake}$ denote the advantage that an adversary breaks the long-term secret key within time t*_4_. *Then*, *the probability that an adversary breaks the AKE security of the DH-3AKA-MA protocol*:

$$Adv_{dh-3aka-ma}^{ake}(t^{\prime },q_{\text{0}},q_{1},q_{2},q_{3})\leq 2Adv_{dh-3aka}^{ake}(t_{3},q_{\text{0}},q_{1},q_{2},q_{3})+\frac{q_{3}{}^{2}}{2^{l-1}},$$

*where t*′ ≤ *t*_3_ *+ (q*_0_ + *q*_1_ + *q*_2_)⋅*t*_*relay*_ + 2⋅*τ*_2_ + 4⋅*τ*_3_; *the used parameters are defined as in Theorems 7; q*_3_ *denotes the numbers of the Hash queries involving A and B; t*_*relay*_ *is the time of relay a query; τ*_2_ *is the time of generating a random number; and τ*_3_ *is the time to perform an exponential computation*.

The following theorem shows that the proposed DH-3AKA-MA protocol has MA security if the used hash function is secure and the DH-3AKA protocol has AKE security.

**Theorem 9**. Let $Adv_{dh-3aka}^{ake}$ *denote the advantage an adversary breaks the AKE security of the DH-3AKA protocol within time t*_4_. *Let $Adv_{dh-3aka-ma}^{ma}$ denote the advantage in violating the explicit mutual authentication of the DH-3AKA-MA protocol. Then, we have*

$$\begin{array}{l} Adv_{dh-3aka-ma}^{ma}(t^{\prime },q_{\text{0}},q_{\text{1}},q_{\text{2}},q_{3})\leq \\ \;2Adv_{dh-3aka}^{ake}(t_{4},q_{\text{0}},q_{\text{1}},q_{\text{2}})+\frac{q_{3}{}^{2}+1}{2^{l-1}} \end{array}$$

*where t*′ ≤ *t*_4_ *+ (q*_0_ + *q*_1_ + *q*_2_)⋅*t*_*relay*_ + 2⋅*τ*_2_, *the used parameters are defined as in Theorems 9 and 10*.

### Performance analyses and comparisons

Table 3 shows a performance comparison of the related 3AKA-MA protocol and the 3AKA-MA protocols proposed here, where *T*_*E*_ denotes the time to execute a exponential operation; *T*_*AS*_ denotes the time to execute an asymmetric en/decryption operation; *T*_C_ denotes the time required to execute a Chebyshev chaotic map operation; *T*_*S*_ denotes the time to execute a symmetric en/decryption operation, and *T*_*H*_ denotes the time to execute a hash operation.

**Table 3:** Comparison of the related 3PAKA protocols and the proposed protocols.

Protocols	P_1_	P_2_	P_3_	P_4_	P_5_	P_6_
Gong′s 3PAKA-MA [2,3]	N	8*T*_*S*_+2*T*_*H*_	N	5m/4r	Y	Y
Amin et al.′s 3AKA-MA [16]	N	32*T*_*H*_	N	6m/-	Y	N
Amin-Biswas′s 3AKA [40]	N	20*T*_*H*_	N	4m/-	N	Y
Proposed 3PAKA-MA	N	8*T*_*S*_+2*T*_*H*_	N	5m/4r	Y	Y
Lin et al.′s DH-3AKA-MA [9]	Y	6*T*_*E*_+6*T*_*S*_+4*T*_*H*_	Y	5m/-	Y	N
Lu and Cao′s DH-3AKA [12]	Y	10*T*_*E*_+8*T*_*MD*_+10*T*_*H*_	Y	5m/4r	N	N
Lee et al.′s DH-3AKA-MA [15]	Y	11*T*_*C*_+8*T*_*S*_+12*T*_*H*_	Y	5m/-	Y	N
Lee et al.′s DH-3AKA-MA [25]	Y	10*T*_*E*_+16*T*_*H*_	Y	5m/-	Y	N
Lee et al.′s DH-3AKA-MA [35]	Y	4 *T*_*AS*_+4 *T*_*C*_+4*T*_*H*_	Y	4m/3r	Y	Y
Lee et al.′s DH-3AKA [36]	Y	8*T*_*C*_+11*T*_*H*_	Y	4m/-	N	Y
Li et al.′s DH-3AKA-MA [37]	Y	4*T*_*C*_+8*T*_*S*_ +11*T*_*H*_	Y	5m/-	Y	N
Proposed DH-3AKA-MA	Y	4*T*_*E*_+8*T*_*S*_+4*T*_*H*_	Y	4m/3r	Y	Y

P_1_: Revealing sub-keys; P_2_: Computational cost; P_3_: Providing perfect forward secrecy;

P_4_: Transmissions; P_5_: Providing explicit mutual authentication; P_6_: Realizing lower bounds.

The first comparison item lists whether or not the sub-keys for generating a session key can be revealed in the channel. In Gong′s protocol [2, 3], Amin et al.′s protocol [16], Amin and Biswas′s protocol [41] and the proposed protocols, the sub-keys cannot be revealed in the channel and clients must exchange their sub-key via the help of the server. In related DH-3AKA-MA protocols and the proposed DH-3AKA-MA protocols, session key security is based on the Diffie-Hellman problem, and thus clients can directly exchange their sub-keys.

The second comparison item is computational cost. Gong′s 3AKA-MA protocol, Amin et al.′s 3AKA-MA [16], Amin and Biswas′s protocol [41] and the proposed 3AKA-MA protocols only require symmetric en/decryption and hash processes, but do not provide perfect forward secrecy. The related DH-3AKA-MA protocols [9,12,15,25,35–37] require more exponential computations or Chebyshev chaotic map operations. The proposed DH-3AKA-MA protocols also require eight en/decryption processes and four exponential computations. Although extra modular exponential costs or Chebyshev chaotic map operations are required, the related DH-3AKA-MA protocols and the proposed DH-3AKA-MA protocols provide perfect forward secrecy.

The number of transmissions was also compared. Gong′s 3AKA-MA protocol and the proposed 3AKA-MA protocols require five messages and four rounds. These protocols thus realize the lower bounds on communications for 3AKA-MA protocols without revealing sub-keys in the channel. Although Amin and Biswas 3AKA [40] protocol requires fewer messages than other protocols, but does not provide explicit mutual authentication. In addition, the related DH-3AKA-MA protocols in [35] and [36] and the proposed DH-3AKA-MA protocol requires four messages and three rounds. However, Lee et al.′s DH-3AKA-MA protocol [35] require server public keys, thus is inefficient in computations. Lee et al.′s DH-3AKA-MA protocol [36] only requires four messages, but does not provide explicit mutual authentication. Altogether, the proposed protocols involve fewer transmissions than other 3AKA-MA protocols, and realize the lower bounds on communications for 3AKA-MA protocols.

## Conclusions

This investigation has provided the lower bounds on communications for 3AKA-MA protocols. In addition, it also considered the lower bounds on communications for the 3AKA-MA protocols whose sub-keys cannot be revealed in the channel and for the 3AKA-MA protocols in which revealing the sub-keys cannot compromise the session key. By using the derived results for the lower bounds on communications, communication-efficient and provably secure 3AKA-MA protocols were developed. As seen in Table 3, the proposed 3AKA-MA protocols involve fewer transmissions than other related 3AKA-MA protocols, but also realize the newly defined lower bounds on communications for 3AKA-MA protocols and are suitable for practical environments. Therefore, a 3AKA-MA protocol, which is developed by using the derived results in this paper, involves fewer transmissions and is efficient in communication.

## Appendix A

**Proof of Theorem 3:** In a 3AKA-MA protocol, by Rule 1, the protocol originator A initiates a message, the other participants B and S issue messages at the moment of receiving one, and the protocol proceeds in sequential order. Then, by using similar arguments in Theorem 2, we have many variable protocols, as shown in Fig 2.

For protocol (a), after the first message, B can receive a sub-key *K*_1_ from A and derive the session key SK by Rule 3. Then, by Rule 4, B can compute and issue an authenticator *auth*_*B*_ in the second message. On the other hand, after the third message, A can authenticate B via S by Rule 6, receive a sub-key *K*_2_ from B, derive the session key SK by Rule 3, and verify *auth*_*B*_ from B. Then, by Rule 4, A can compute and issue an authenticator *auth*_*A*_ in the fourth message. Finally, B can authenticate A via S by Rule 6 and verify *auth*_*A*_ from A. Hence, for A and B, four messages are required. In addition, for S, three messages are required by Rules 1 and 2. Therefore, the 3AKA-MA protocol can be implemented in four messages in (a).

For protocol (b), after the third message, A can receive a sub-key *K*_2_ from B and derive the session key SK by Rule 3. By Rule 4, A can compute and issue an authenticator *auth*_*A*_ in the fourth message. However, by Rule 5, B must receive and verify *auth*_*A*_ from A, and thus requires an extra message at least. Therefore, protocol (b) cannot be implemented in four messages.

For protocol (c), by Rules 3, A cannot derive the session key SK until it receives a sub-key *K*_2_ from B. Thus, for A, an extra message is required to receive the *K*_2_. In addition, another extra message is required to issue an authenticator *auth*_*A*_. Hence, at least two extra messages are required by Rules 4 and 5. Thus protocol (c) cannot be implemented in four messages.

For protocol (d), by Rule 3, A can obtain a sub-key *K*_2_ from B and derive the session key SK after it receives a message from B in the second message. Then, by Rule 4, A can compute and issue an authenticator *auth*_*A*_ in the third message. However, by Rule 5, B must receive and verify *auth*_*A*_ from A, and thus requires an extra message at least. Therefore, protocol (d) cannot be implemented in five messages.

For protocol (e), by Rule 6, A cannot authenticate B via S until it receives a message sent from B and transmitted by S. Then, A requires an extra message at least. Therefore, protocol (e) cannot be implemented in four messages.

For protocol (f), by Rules 1 and 2, S can issue messages only while receiving one and must send out a message. Therefore, two extra messages are required at least. Thus protocol (f) cannot be implemented in four messages.

For protocol (g), by Rule 3, A cannot derive the session key SK until it receives a sub-key *K*_2_ from B. Then, A requires an extra message to receive the sub-key *K*_2_ from B. In addition, by Rules 4 and 5, another extra one is required to issue an authenticator *auth*_*A*_. Therefore, protocol (g) requires two extra messages at least, and thus cannot be implemented in four messages.

For protocol (h), by Rule 6, A cannot authenticate B via S since it does not receive a message sent from B and transmitted by S. Therefore, protocol (h) requires two extra messages at least, and thus is implemented in at least six messages.

For protocol (i), by Rule 3, A can receive a sub-key *K*_2_ from B and derive the session key SK after receiving a message from B in the third message. Then, by Rule 4, A can issue an authenticator *auth*_*A*_ in the fourth message. However, by Rule 5, B must receive and verify *auth*_*A*_, and thus requires an extra message at least. Therefore, protocol (i) cannot be implemented in four messages.

For protocol (j), by Rules 1 and 2, B can issue messages only while receiving one and must send out a message including a sub-key *K*_2_. Then, for B, two extra messages are required at least. On the other hand, A can derive the session key SK after receiving a sub-key *K*_2_ from B. In addition, A requires an extra message to issue an authenticator *auth*_*A*_ by Rules 4 and 5. Hence, three extra messages are required at least. Thus the protocol (j) cannot be implemented in four messages.

Table II summarizes the analyses of protocols (a),(b),…,(j). From these analyses, we can conclude that with the exceptions of protocol (a), these 3AKA-MA protocols cannot be implemented in four messages. Therefore, every 3AKA-MA protocol requires at least four messages for implementation.

**Proof of Theorem 6:** Assume that *P* and *Q* range over principals. *C* denotes a communicating channel and *X* and *Y* are messages. The followings define the notation used for logical analyses.

*C*(*X*): The message *X* is transited via channel *C*.
*r*(*C*): The set of readers of channel *C*.
*w*(*C*): The set of writers of channel *C*.
*P* ⊲ *C*(*X*): *P* sees *C*(*X*). The message *X* is transited via channel *C* and can be observed by *P*. *P* must be a reader of channel *C* to read message *X*.
*P* ⊲ *X*|*C*: *P* sees *X* via *C*. The message *X* is transited via channel *C* and can be received by *P*.

The used assumptions and logic rules [38,39,42] and the logical description of the proposed protocol are describes as follows.

The Assumptions used in [38,39,42], where *U* and *V* are *S*, *A* and *B*, and *U* ≠ *V*:

(A1) *U* ∈ *r*(*C*_*U*,*V*_): *U* can read from the channel *C*_*U*,*V*_.
(A2) *U* ≡ (*w*(*C*_*U*,*V*_) = {*U*,*V*}: *U* believes that *U* and *V* can write on *C*_*U*,*V*_.
(A3) *U* ≡ (*V*∥~Φ → Φ): *U* believes that *V* only says what it believes
(A4) *U* ≡ #(*N*_*U*_): *U* believes that *N*_*U*_ is fresh.

The Inference Rules of the Logic:

**Seeing rules**
(S1) $\frac{P⊲C(X),P\in r(C)}{P\equiv (P⊲X|C),P⊲X}$: If *P* receives and reads *X* via *C*, then *P* believes that *X* has arrived on *C* and *P* sees *X*.
(S2) $\frac{P⊲(X,Y)}{P⊲X,P⊲Y}$: If *P* sees a hybrid message (*X*, *Y*), then *P* sees *X* and *Y* separately.

**Interpretation rules**
(I1) $\frac{P\equiv (w(C)=\{P,Q\})}{P\equiv (P⊲X|C)\to Q|\sim X}$: If *P* believes that *C* can only be written by *P* and *Q*, then *P* believes that if *P* receives *X* via *C*, then *Q* said *X*.
(I2) $\frac{P\equiv (Q|\sim (X,Y))}{P\equiv (Q|\sim X),P\equiv (Q|\sim Y)}$: If *P* believes that Q said a hybrid message (*X*, *Y*), then *P* believes that *Q* has said *X* and *Y* separately.

**Freshness rules**
(F1) $\frac{P\equiv (Q|\sim X),P\equiv \#(X)}{P\equiv (Q||\sim X)}$: If *P* believes that another *Q* said *X* and *P* also believes that *X* is fresh, then *P* believes that *Q* has recently said *X*.
(F2) $\frac{P\equiv \#(X)}{P\equiv \#(X,Y)}$: If *P* believes that a part of a mixed message *X* is fresh, then it believes that the whole message (*X*,*Y*) is fresh.

**Rationality rules**
(R1) $\frac{P\equiv (\Phi_{1}\to \Phi_{2}),P\equiv \Phi_{1}}{P\equiv \Phi_{2}}$: If *P* believes that Φ_1_ implies Φ_2_ and *P* believes that Φ_1_ is true, then *P* believes that Φ_2_ is true.

According to the logic in [38, 39, 42], the proposed protocol is described as follows.

Step 1 *S* ⊲ (*A*, *B*, *C*_*S*,*A*_(*A*, *S*, *A*, *K*_1_))
  *B* ⊲ (*A*, *B*)
Step 2 *S* ⊲ (*A*, *B*, *C*_*S*,*B*_(*B*, *S*, *B*, *K*_2_))
Step 3 *A* ⊲ (*C*_*S*,*A*_(*S*, *A*, *B*, *K*_2_))
  *B* ⊲ (*C*_*S*,*B*_(*S*, *B*, *A*, *K*_1_))
Step 4 *B* ⊲ (*C*_*A*,*B*_(*A*, *B*, *K*_1_, *K*_2_))
  *A* ⊲ (*C*_*A*,*B*_(*B*, *A*, *K*_1_, *K*_2_))

According the assumptions and logical analyses, the proposed protocol must realize the goals of authentication and key agreement:

Goal 1: $A\;\equiv \;A\begin{array}{c} \underset{\leftarrow \to }{f(K_{1},K_{2})} \end{array}B$: Participant *A* believes that *SK* = *f*(*K*_1_, *K*_2_) is a symmetric key shared between participants *A* and *B*.

Goal 2: *$B\;\equiv \;A\begin{array}{c} \underset{\leftarrow \to }{f(K_{1},K_{2})} \end{array}B$*: Participant *B* also believes that *SK* = *f*(*K*_1_, *K*_2_) is a symmetric key shared between participants *A* and *B*.

Goal 3: *$A\;\equiv \;B\;\equiv \;A\begin{array}{c} \underset{\leftarrow \to }{f(K_{1},K_{2})} \end{array}B$*: Participant *A* believes that *B* is convinced of *SK* = *f*(*K*_1_, *K*_2_) is a symmetric key shared between participants *A* and *B*.

Goal 4: *$B\;\equiv \;A\;\equiv \;A\begin{array}{c} \underset{\leftarrow \to }{f(K_{1},K_{2})} \end{array}B$*: Participant *B* also believes that *A* is convinced of *SK* = *f*(*K*_1_, *K*_2_) is a symmetric key shared between participants *A* and *B*.

For achieving Goal 1, we have that

Sub-goal 1–1: *A* ≡ *K*_1_ by using the interpretation rule (I3)),

Sub-goal 1–2: *A* ≡ *K*_2_ by using the rationality rule (R1),

Sub-goal 1–3: *A* ≡ (*S*∥~*C*_*S*,*B*_(*B*, *S*, *B*, *K*_2_) → *C*_*S*,*B*_(*B*, *S*, *B*, *K*_2_)) by using assumption (A3)) and Sub-goal 1–4: *A* ≡ (*S*∥~*K*_2_).

Then, we have that

Sub-goal 1–5: *A* ≡ #(*K*_2_) by using the freshness rules (F1, F2) and assumption (A11)),

Next, we use the interpretation rule (I1) and the seeing rule (S1), and have that

Sub-goal 1–6: *A* ∈ *r*(*C*_*S*,*A*_) by using assumption (A1),

Sub-goal 1–7: *A* ≡ (*w*(*r*(*C*_*S*,*A*_) = {*A*, *S*}) by using assumption (A3) and

Sub-goal 1–8: *A* ≡ ⊲ *C*_*S*,*A*_(*K*_2_) by using the seeing rule (S2).

Thus, the proposed protocol provides Goal 1: $A\;\equiv \;A\begin{array}{c} \underset{\leftarrow \to }{f(K_{1},K_{2})} \end{array}B$.

Similarly, using the same derivation of Goal 1, we have that the proposed protocol provides Goal 2: *$B\;\equiv \;A\begin{array}{c} \underset{\leftarrow \to }{f(K_{1},K_{2})} \end{array}B$*.

For achieving Goal 3, we have that

Sub-goal 3–1: $A\;\equiv \;((B||\sim A\begin{array}{c} \underset{\leftarrow \to }{f(K_{1},K_{2})} \end{array}B)\to (B\equiv A\begin{array}{c} \underset{\leftarrow \to }{f(K_{1},K_{2})} \end{array}B))$ by using the rationality rule (R1) assumption (19)) and

Sub-goal 3–2: $A\;\equiv \;(B||\sim A\begin{array}{c} \underset{\leftarrow \to }{f(K_{1},K_{2})} \end{array}B)$.

Then, we have that

Sub-goal 3–3: $A\;\equiv \;(B|\sim A\begin{array}{c} \underset{\leftarrow \to }{f(K_{1},K_{2})} \end{array}B)$ by using the freshness rule (F1) and

Sub-goal 3–4: $A\;\equiv \#(A\begin{array}{c} \underset{\leftarrow \to }{f(K_{1},K_{2})} \end{array}B)$ by using the freshness rule (F2) and assumption (A11)).

Next, we use the interpretation rule (I1) and the seeing rule (S1), and have that

Sub-goal 3–5: *A* ∈ *r*(*C*_*A*,*B*_) by using assumption (A15),

Sub-goal 3–6: *A* ≡ (*w*(*C*_*A*,*B*_) = {*A*, *B*}) by using assumption (A17) and

Sub-goal 3–7: $A⊲C_{A,B}(A\begin{array}{c} \underset{\leftarrow \to }{f(K_{1},K_{2})} \end{array}B)$ by using the seeing rule (S2).

Thus, the proposed protocol provides Goal 3: *$A\;\equiv \;B\;\equiv \;A\begin{array}{c} \underset{\leftarrow \to }{f(K_{1},K_{2})} \end{array}B$*.

Similarly, using the same arguments of Goal 3, the proposed protocol provides Goal 4: *$B\;\equiv \;A\;\equiv \;A\begin{array}{c} \underset{\leftarrow \to }{f(K_{1},K_{2})} \end{array}B$*.

Then the proof is concluded.

**Proof of Theorem 7:** Using similar arguments in [35], the proof also consists of a sequence of games starting at the game ${\text{G}}_{0}^{ake}$. The first game is the real attack against the DH-3AKA protocol and the terminal game ${\text{G}}_{4}^{ake}$ concludes that the adversary has a negligible advantage to break the AKE security of the DH-3AKA protocol.

**Game ${\text{G}}_{0}^{ake}$**: This game corresponds to the real attack. By definition, we have

$$Adv_{dh-3aka}^{ake}(A_{\text{ake}})=|2\text{Pr}[E_{\text{0}}]-1|.$$ (1)

The following games ${\text{G}}_{1}^{ake}$ and ${\text{G}}_{2}^{ake}$ can be derived by using similar arguments of Theorem 5.2.

**Game ${\text{G}}_{1}^{ake}$**: This game simulates all oracles as in previous game except for replacing the long-term secret keys with two random numbers. Then, we have

$$|\text{Pr}[E_{\text{0}}]-\text{Pr}[E_{\text{1}}]|\leq 2\cdot Adv_{sk}(A_{\text{1}}).$$ (2)

**Game ${\text{G}}_{2}^{ake}$**: This game simulates all oracles as in previous game except for using two table lists to simulate ***SymEnc*** queries. Then, we have

$$|\text{Pr}[E_{\text{1}}]-\text{Pr}[E_{2}]|\leq \frac{q_{\text{1}}^{2}+q_{2}^{2}}{2^{l}},$$ (3)

**Game ${\text{G}}_{3}^{ake}$**: This game simulates all oracles as in previous game except for modifying the simulation of ***Send*** queries refereeing the flows containing *g*^*x*^ in Step 1 and *g*^*y*^ in Step 2 of the DH-3AKA protocol and the simulation of the **Test**(*U*^*i*^) oracle to avoid relying on the knowledge of *x*, *y* and *z* used to compute the answer to these queries. Assume that (*X* = *g*^*x*^, *Y* = *g*^*y*^, *Z* = *g*^*xy*^) is a random DDH triple. Using similar arguments in [35], we have that the set of random variables in ${\text{G}}_{2}^{ake}$ is replaced by another set of identically distributed random variables in ${\text{G}}_{3}^{ake}$. ${\text{G}}_{2}^{ake}$ is equivalent to ${\text{G}}_{3}^{ake}$ and

$$\text{Pr}[E_{2}]=\text{Pr}[E_{3}].$$ (4)

**Game ${\text{G}}_{4}^{ake}$**: This game simulates all oracles as in previous game except that all rules are computed using a triple (*X*, *Y*, *Z*) sample from a random distribution (*g*^*x*^, *g*^*y*^, *g*^*z*^), instead of a DDH triple. Using similar arguments in [35], we have

$$\left|\text{Pr}[E_{3}]-\text{Pr}[E_{4}]\right|\leq Adv_{\text{G}}^{ddh}(A_{\text{ddh}}),$$ (5)

and the probability Pr[*E*_4_] is exactly $\frac{1}{2}$.

Combining Eqs (1), (2), (3), (4) and (5), we have

$$Adv_{dh-3aka}^{ake}(A_{\text{ake}})\leq \frac{q_{\text{1}}{}^{2}+q_{2}{}^{2}}{2^{l-1}}\;+4\cdot Adv_{sk}(A_{1})+2\cdot Adv_{G}^{ddh}(A_{\text{ddh}}).$$

Then the proof is concluded.

**Proof of Theorem 8:** The proof also consists of a sequence of games starting at the game ${\text{G}}_{0}^{ake}$. Each game ${\text{G}}_{i}^{ake}$ defines the probability of the event *E*_*i*_ that the adversary wins this game, i.e. *c*' = *c*. The first game is the real attack against the DH-3AKA-MA protocol and the terminal game ${\text{G}}_{3}^{ake}$ concludes that the adversary has a negligible advantage to break AKE security of the DH-3AKA-MA protocol. Assume that the challenger $A_{2}$ attempts to break AKE security of the DH-3AKA protocol, and the adversary $A_{\text{ake}}$ is constructed to break AKE security of the DH-3AKA -MA protocol. The challenger $A_{2}$ returns the real session key *SK* or a random string to $A_{\text{ma}}$ by flipping an unbiased coin *c* ∈ {0,1}. The adversary $A_{\text{ma}}$ wins if it correctly guesses bit *c*.

The following game models that $A_{\text{ake}}$ tries to distinguish the real session key from the random string.

**Game ${\text{G}}_{0}^{ake}$**: This game corresponds to the real attack. By definition, we have

$$Adv_{3aka\text{1-}ma}^{ake}(A_{\text{ake}})=|2\text{Pr}[E_{\text{0}}]-1|.$$ (6)

**Game ${\text{G}}_{1}^{ake}$**: This game simulates all oracles as in previous game except for using a table list **H** to simulate ***Hash*** queries involving *A* and *B*. Then, we have

$$|\text{Pr}[E_{\text{0}}]-\text{Pr}[E_{\text{1}}]|\leq \frac{q_{3}^{2}}{2^{l}},$$ (7)

where $A_{\text{ma}}$ makes *q*_3_ ***Hash*** queries involving *A* and *B*.

**Game ${\text{G}}_{2}^{ake}$**: This game simulates all oracles as in previous game except for replacing the session key *SK* with a random number. Then, we can use $A_{\text{ma}}$ to build an adversary $A_{2}$ against the AKE security of DH-3AKA. First, $A_{2}$ sets up the parameters, starts simulating the DH-3AKA -MA protocol and answers the oracle queries made by $A_{\text{ma}}$ as follows.

- When $A_{\text{ma}}$ make ***Send*** or ***SymEnc*** queries, $A_{2}$ answers what the DH-3AKA protocol says to.
- When $A_{\text{ma}}$ makes ***Hash*** queries, $A_{2}$ answers corresponding authenticators to $A_{\text{ma}}$ by making the same queries to the oracle Hash.
- When $A_{\text{ma}}$ makes ***Test*** queries, $A_{2}$ answers these queries using the bit *c* that it has previously selected and the session keys that has computed.

Accordingly, the probability that $A_{2}$ outputs 1 when its Test oracle returns the real authentication keys is equivalent to the probability that $A_{\text{ake}}$ correctly guesses the hidden bit *c* in game ${\text{G}}_{1}^{ake}$. Similarly, the probability that $A_{2}$ outputs 1 when its Test oracle returns the random strings is equivalent to the probability that $A_{\text{ma}}$ correctly guesses the hidden bit *c* in game ${\text{G}}_{2}^{ake}$. Thus, by Lemma 1, we have

$$\left|\text{Pr}[E_{\text{1}}]-\text{Pr}[E_{2}]|\leq Adv_{dh-3aka}^{ake}(A_{2}\right)$$ (8)

At this time, no information on the hidden bit *c* is leaked to the adversary. It is straightforward that

$$\text{Pr}[E_{2}]=\frac{1}{2}.$$ (9)

Combining Eqs (6), (7), (8) and (9), we have

$$Adv_{\text{dh-3}aka-ma}^{ake}(A_{\text{ma}})\leq 2Adv_{dh-3aka}^{ake}(A_{2})+\frac{q_{3}{}^{2}}{2^{l-1}}.$$

Then the proof is concluded.

**Proof of Theorem 9:** The proof also consists of a sequence of games starting at the game ${\text{G}}_{0}^{ma}$. The first game is the real attack against the DH-3AKA-MA protocol and the terminal game ${\text{G}}_{3}^{ma}$ concludes that the adversary has a negligible advantage to break MA security of the DH-3AKA protocol. The challenger $A_{3}$ attempts to break MA security for the DH-3AKA protocol and the adversary $A_{\text{ma}}$ is constructed to break MA security for the DH-3AKA-MA protocol. The adversary $A_{\text{ma}}$ wins this game if he successfully fakes the authenticator *μ*_*A*_ or *μ*_*B*_.

**Game ${\text{G}}_{0}^{ma}$**: This game corresponds to the real attack. By definition, we have

$$Adv_{dh-3aka-ma}^{ma}(A_{\text{ma}})=2\text{Pr}[E_{\text{0}}].$$ (10)

**Game ${\text{G}}_{1}^{ma}$**: Similar to ${\text{G}}_{1}^{ake}$ in Theorem 8, this game simulates all oracles as in previous game except for using a table list **H** to simulate ***Hash*** queries involving *A* and *B*. Then, we have

$$|\text{Pr}[E_{\text{0}}]-\text{Pr}[E_{\text{1}}]|\leq \frac{q_{3}^{2}}{2^{l}},$$ (11)

where $A_{\text{ma}}$ makes *q*_3_ ***Hash*** queries involving *A* and *B*.

**Game ${\text{G}}_{2}^{ma}$**: This game simulates all oracles as in previous game except for replacing the session key *SK* with a random number. Then, we can use $A_{\text{ma}}$ to build an adversary $A_{3}$ against the AKE security of 3AKA1. Using similar arguments for ${\text{G}}_{2}^{ake}$ in Theorem 8, we have

$$\left|\text{Pr}[E_{\text{1}}]-\text{Pr}[E_{2}]|\leq Adv_{dh-3aka}^{ake}(A_{3}\right)$$ (12)

No information on the authenticator is leaked to the adversary, and thus

$$\text{Pr}[E_{2}]=\frac{1}{2^{l}}.$$ (13)

Combining Eqs (10), (11), (12) and (13), we have

$$Adv_{dh-3aka}^{ake}\;(A_{\text{ma}})\;\leq 2Adv_{dh-3aka}^{ake}(A_{3})+\frac{q_{3}{}^{2}+1}{2^{l-1}}.$$

Then the proof is concluded.
